# Immune, blood-brain barrier, and metabolic biomarkers mediate gut-brain axis crosstalk in alzheimer’s disease

**DOI:** 10.1186/s40364-025-00851-6

**Published:** 2025-10-29

**Authors:** Jincheng Li, Ziyu Yuan, Jialin Li, Zhenqiu Liu, Yingzhe Wang, Mei Cui, Chen Suo, Li Jin, Ding Ding, Xingdong Chen, Yanfeng Jiang

**Affiliations:** 1 State Key Laboratory of Genetics and Development of Complex Phenotypes, Human Phenome Institute, Research and Innovation Center, Shanghai Pudong Hospital, Zhangjiang Fudan International Innovation Center, and National Clinical Research Center for Aging and Medicine, Fudan University, Shanghai, 200433 China; 2 Fudan University Taizhou Institute of Health Sciences, Taizhou, 225300 Jiangsu China; 3 Department of Neurology, Huashan Hospital, Fudan University, Shanghai, 200040 China; 4 Ministry of Education Key Laboratory of Public Health Safety, Department of Epidemiology, School of Public Health, Fudan University, Shanghai, 200032 China; 5 Institute of Neurology, National Clinical Research Center for Aging and Medicine, National Center for Neurological Disorders, Huashan Hospital, Fudan University, Shanghai, 200040 China

**Keywords:** Gut–Brain axis, Alzheimer’s disease, Gut microbiota, Circulating Immune–Blood–Brain Barrier–Metabolic biomarkers, Mendelian randomization

## Abstract

**Background:**

Gut microbiota may influence Alzheimer’s disease (AD) pathogenesis by modulating host homeostasis. However, population-based causal evidence linking gut dysbiosis to Alzheimer’s disease pathogenesis, especially via immune, vascular, and metabolic pathways, remains insufficient.

**Methods:**

We performed Mendelian randomization (MR) and colocalization analysis on 629 gut microbiota features and 2,103 immune, blood-brain barrier (BBB), and metabolic biomarkers regarding the risk of AD and cerebrospinal fluid (CSF) pathological biomarkers.

**Results:**

We identified that mucin-degraders, short-chain fatty acid (SCFA) producers, and Programmed Cell Death Protein 1/Programmed Death-Ligand 1 (PD-1/PD-L1)-related biomarkers were associated with lower AD risk, while cardiovascular microbes, Amyloid-beta (Aβ)-related proteins, and lipoproteins were linked to higher risk. Increased AD risk was associated with decreased SCFA producers, branched-chain amino acids (BCAAs), and lactate, but with increased liver-disease microbes, fatty acids, and glycoprotein acetyls. Notably, *Desulfovibrionaceae* and *Methanobrevibacter* emerged as critical contributors to AD. *Erysipelotrichaceae* abundance inversely modulates CSF phosphorylated tau (p-tau) pathology while being increased by Aβ42 pathology, suggesting a microbiota-mediated feedback circuit in AD. Mediation analysis highlighted the role of CD28^−^CD8^+^ T cells, CD19 on IgD^+^ CD24^+^ B cells, glycoproteins, and low-density lipoprotein (LDL) in microbiota-gut-brain axis bidirectional communication. Colocalization analyses confirmed causal links between AD and LDL metabolism through shared variant rs7412 (posterior probability, PP = 1.0), while revealing colocalized architecture for amyloid-tau copathology at rs71352238 (PP = 1.0).

**Conclusions:**

Our study reveals a bidirectional gut–brain feedback loop in AD, in which gut microbiota promote neuroinflammation and immune aging, while AD exacerbates gut dysbiosis via lipid metabolic dysregulation. This self-reinforcing mechanism involving immune signaling, BBB disruption, and SCFA imbalance offers potential targets for integrated microbiota-based interventions in AD prevention.

**Supplementary Information:**

The online version contains supplementary material available at 10.1186/s40364-025-00851-6.

## Introduction

 Alzheimer’s Disease (AD) is a progressive neurodegenerative disorder with increasing prevalence in current aging societies. It follows a continuous pathophysiological process [1], with pathologic changes beginning 15 to 20 years before the onset of clinical symptoms [2, 3]. Despite extensive research, our understanding of the mechanisms beyond the aggregation of the amyloid-β (Aβ) plaques, tau protein neurofibrillary tangles (NFTs), neuroinflammation, and brain atrophy remains limited, hampering the development of effective prevention and treatment strategies for AD [4].

Recent evidence increasingly suggests that the gut microbiota, as a component of the microbiota-gut-brain axis, plays a crucial role in the development of AD [5–7]. Even in the preclinical stage of AD, alterations in the gut microbiota are significantly linked to the formation of Aβ plaques and tau tangles [8]. This emerging connection between gut microbiota and brain pathophysiology underscores the need for a deeper investigation into how the microbiota-gut-brain axis contributes to AD pathogenesis, unveiling new insights into its etiology. Metabolites produced by the gut microbiota have been detected in both blood and cerebrospinal fluid (CSF) [9] and linked to key pathologies of AD [10, 11]. Accumulating evidence from mechanistic investigations has established that immune signaling, blood-brain barrier integrity, and systemic metabolism integrate to form the microbiota-gut-brain axis, with emerging preclinical and clinical evidence implicating this axis in gut microbiota-Alzheimer’s disease pathogenesis [12, 13]. However, due to restricted cohort dimensions, prohibitive costs of next-generation sequencing and AD pathological phenotyping, longitudinal follow-up limitations, and ethical constraints in human studies, the ability to establish causality and confirm the mechanistic links between gut microbiota and AD at the population level remains limited [14]. Translational animal model studies have advanced this field and demonstrated that the gut microbiota can influence AD pathologies by modulating the integrity of the blood-brain barrier (BBB), as well as through peripheral immune and metabolic pathways, providing causal evidence of the relationship between gut microbiota and AD [15–18]. Nevertheless, translational gaps stemming from the inherent differences between model organisms and humans in dietary patterns, environmental exposures, and genetic architectures [14] preclude the definitive establishment of causal relationships and mechanistic underpinnings linking gut microbiota to Alzheimer’s disease pathogenesis.

Mendelian randomization (MR) offers a valuable approach to investigate the causal relationship between gut microbes and AD. By using genetic variants as instrumental variables (IV), MR can infer causal links between exposures and outcomes. Since genetic variants are fixed at conception, they can be considered analogous to a natural randomized controlled trial, helping to minimize confounding and reverse causality [19]. Current MR studies have examined the causal relationships between gut microbiota and AD, exploring the potential roles of blood metabolites, and have demonstrated that cytokines do not serve as mediators [20–22]. Despite evidence supporting the roles of immune signaling, BBB integrity, and systemic metabolism in the microbiota–gut–brain axis, their precise involvement in the causal interplay between gut microbiota and AD remains unclear. To address this gap, we prioritized biomarkers with prior evidence of potential mediating role in the gut–brain axis and restricted our analyses to large-scale GWAS datasets from non-overlapping, ancestrally homogeneous populations to enhance the robustness of causal inference.

**Fig. 1:**
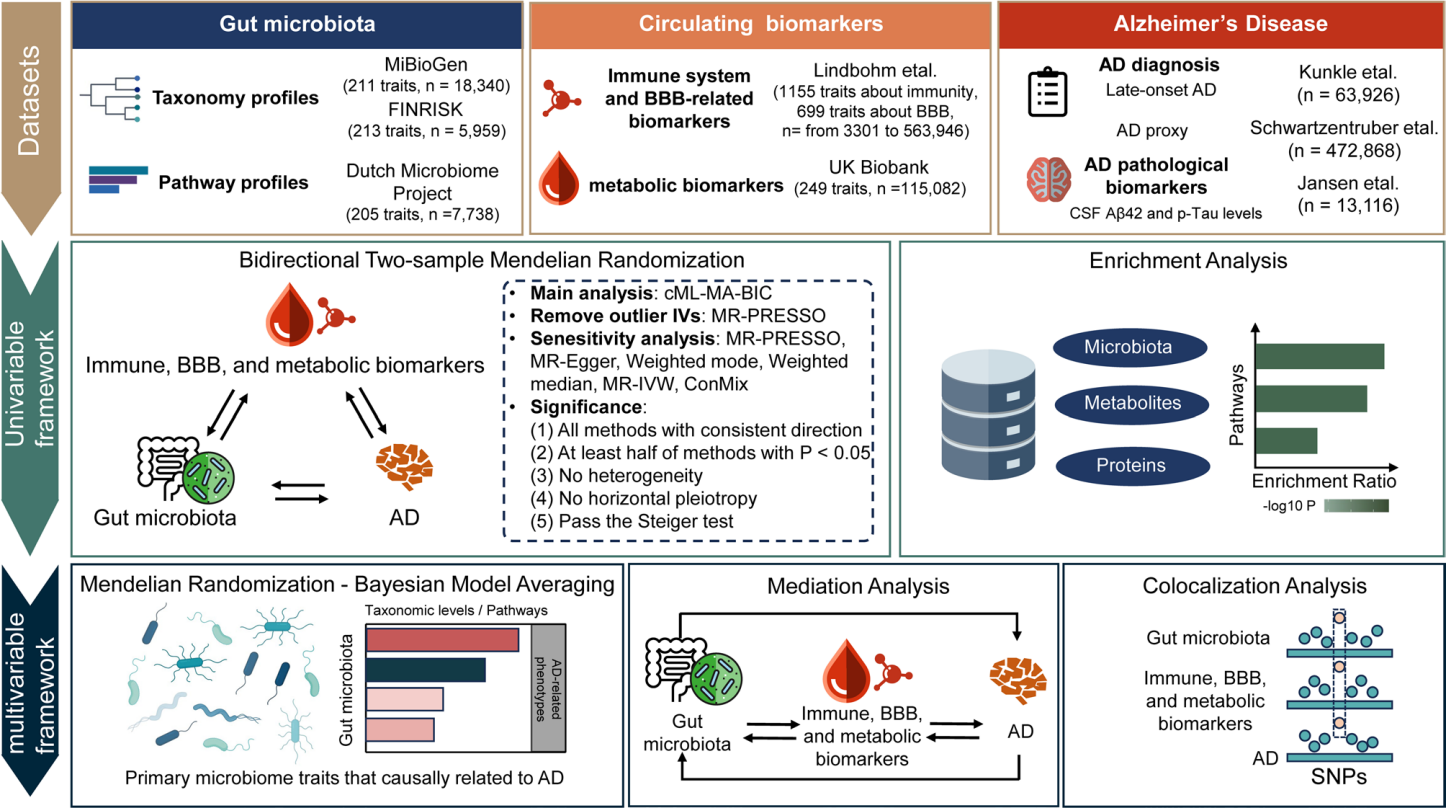
Schematic overview of the study design. GWAS summary data included taxonomy and pathway profiles for gut microbiota, as well as blood metabolites, immune markers, blood-brain barrier(BBB)-related biomarkers, and Alzheimer’s disease (AD) phenotypes, including cerebrospinal fluid (CSF) pathological biomarkers. In the univariable framework, bidirectional two-sample mendelian randomization (MR) was conducted to explore causal relationships, followed by enrichment analysis for biological process annotation. In the multivariable framework, mendelian randomization-Bayesian Model Averaging (MR-BMA) was applied to identify key gut microbiota associated with AD risk. Mediation analysis assessed the intermediary role of circulating biomarkers in the gut microbiota-AD interaction, while colocalization analysis validated the MR findings

In this study, we investigated the hypothesis that immune signaling, BBB integrity, and systemic metabolism contribute to the bidirectional crosstalk between gut microbiota and AD. We conducted systematic MR and colocalization analyses to comprehensively investigate the relationships between gut microbiota, immune, BBB, and metabolic biomarkers, and AD, providing insights from a population-based perspective (Fig. 1). We integrated multi-dimensional, large-scale genome-wide association study (GWAS) summary statistics from diverse sources for both exposure and outcome, employing various MR methods to jointly address potential prodromal and selection biases [23], while comprehensive databases were leveraged to interpret the underlying biological mechanisms.

## Methods and materials

### GWAS summary data

#### Gut microbiota

 Genetic variants for gut microbiota included both taxonomic and pathway profiles, which derived from FINRISK 2002 (FR02) cohort (473 taxa, *N* = 5,959) [24], MiBioGen consortium(211 taxa, *N* = 18,340 [25], and the Dutch Microbiome project (205 pathways, *N* = 7,738) [26].

#### Circulating biomarkers

The GWAS data of immune system and BBB-related traits were sourced from Lindbohm et al. study [27], including 1155 immune-related traits and 699 BBB-related traits (*N* = 3,301 ~ 563,964). Metabolic data were obtained from the UK Biobank (249 metabolic traits, *N* = 115,082) [28].

#### AD phenotype

The GWAS data for AD included both AD diagnosis traits and AD pathological biomarkers. Genetic instruments for AD diagnosis containing late-onset AD(LOAD) (*N* = 63,926) [29], and AD proxy(*N* = 472,868) [30]. The AD proxy phenotype was defined in the UK Biobank based on parental history, with participants reporting at least one parent diagnosed with AD classified as proxy cases and those reporting no parental history of AD or dementia classified as proxy controls [31]. The AD proxy was included to address the limited sample size of LOAD and potential bias arising from the disease stage in case samples [30]. AD pathological biomarkers included CSF levels of Aβ42 and p-tau, with the largest sample size (*N* = 13,116) so far [32].

### Mendelian randomization and sensitivity analyses

As different Mendelian randomization methods are based on different assumptions, the adoption of multiple Mendelian methods helps minimize false positives. Multiple univariable MR (UVMR) methods were performed in the present study including inverse variance weighted (IVW) [33], MR-Egger regression [34], weighted median, weighted model [35], Mendelian randomization pleiotropy residual sum and outlier (MR-PRESSO) [36], Contamination mixture method (ConMix) [37], and constrained maximum likelihood and model averaging (cML-MA) [38]. cML-MA-BIC, with its superior power and error control, served as the primary analysis [38]. Significant associations were identified based on: (1) consistent direction across all methods, (2) *P* < 0.05 in at least half of the methods, including the primary analysis, (3) no evidence of pleiotropy or heterogeneity, and (4) passing the Steiger directionality test (Fig. S1). This strategy balances statistical rigor with discovery potential by requiring cross-method consistency (*P* < 0.05 in ≥ 3 of 6 MR methods) to reduce false positives, and applying Nyholt correction at the MR-BMA prioritization stage, while avoiding excessive conservativeness that may obscure true signals. Detailed information can be found in the Supplementary Method.

We further queried the Genotype-Tissue Expression (GTEx) project (version 8) [39] using the R package xQTLbiolinks [40] to examine whether the colocalized loci represent molecular quantitative trait loci (QTLs) in brain and other tissues.

## Results

### Study design

To verify the hypothesis that immune signaling, BBB integrity, and systemic metabolism underpin the bidirectional crosstalk between gut microbiota and AD, we leveraged comprehensive GWAS datasets comprising 629 gut microbiota taxonomic and functional features, as well as 2,103 immune system, BBB-related, and metabolic biomarkers associated with AD risk and CSF pathologies. Initially, we employed bidirectional two-sample Mendelian randomization to examine the causal relationships among gut microbiota and circulating biomarkers—specifically, metabolic, immune system, and BBB-related biomarkers—and AD, and to assess the consistency of gut microbiota’s contribution to AD progression (Fig. S1). Enrichment analysis was then used to further annotate the biological process involved in AD development. Additionally, we employed MR-Bayesian model averaging (MR-BMA) to identify key microbial taxa contributing to different stages of AD progression. By integrating two-step MR and UVMR within a mediation analysis framework, we identified the role of immune signaling, BBB integrity, and systemic metabolism in mediating bidirectional communication between gut microbiota and AD. MR findings were further investigated by colocalization analysis (Fig. 1).

### Identification of AD-associated gut microbiota

**Fig. 2:**
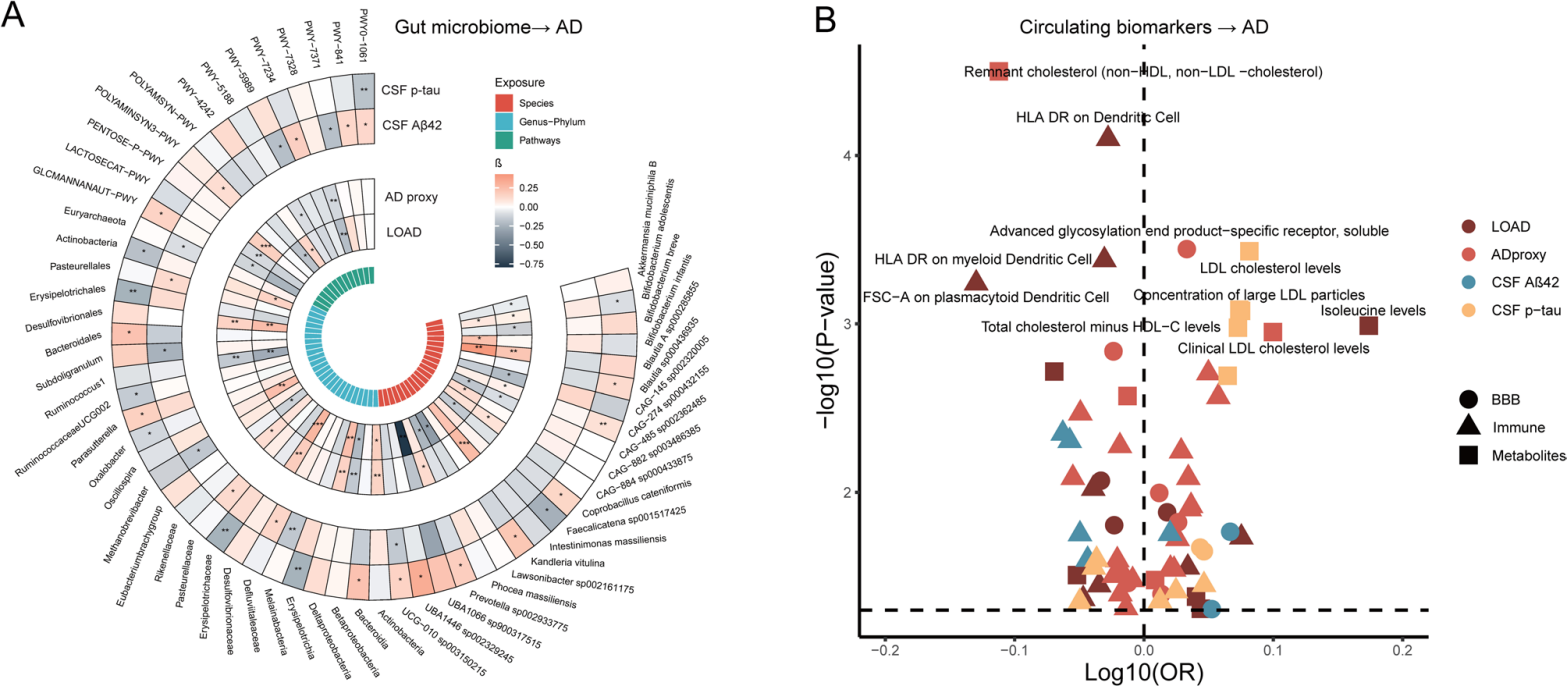
Causal associations between gut microbiota, circulating biomarkers, and Alzheimer’s disease (AD) phenotypes (Gut microbiome/circulating biomarkers → AD). The circular heatmap (**A**) illustrates causal relationships identified in the primary Mendelian randomization analysis using constrained maximum likelihood and model averaging (cML-MA-BIC), showing associations between gut microbiota and AD phenotypes. The inner circle displays associations between gut microbiota and both late-onset AD (LOAD) and AD proxies, while the outer circle highlights associations with CSF pathological biomarkers. Cells are colored by beta coefficients: red for positive and blue for reverse associations. Gut microbiota features are organized by species, genus-phylum, and metabolic pathways. Significance levels are indicated as *P < 0.05; **P < 0.01; ***P < 0.001. The volcano plot (**B**) displays causal relationships between AD and circulating biomarkers, with dot shapes denoting exposure categories and colors representing different AD phenotypes as outcomes. The top 10 significant associations for circulating biomarkers are labeled. Dashed lines indicate *P*-value (*P* < 0.05) and odds ratio (OR = 1) thresholds

A total of 629 features of gut microbiota were tested for causal relationships with four AD-related phenotypes (i.e., LOAD, AD proxy, CSF levels of Aβ42 and p-tau) (Fig. 2A). Using six different UVMR methods and conducting instrument validity tests, we identified 84 species, 100 higher-level taxa (genus to phylum), and 52 metabolic pathways significantly associated with at least one AD-related phenotype (Table S1). At the genus-to-phylum levels, taxa such as *Deltaproteobacteria*, *Desulfovibrionaceae*, and *Desulfovibrionales* were positively associated with LOAD and AD proxy, whereas *Betaproteobacteria*, *Ruminococcus1* were reversely associated. At the species level, *Blautia A* sp000285855, *Prevotella* sp002933775, *CAG-274* sp000432155, and *UCG-010 sp003150215* were positively linked to AD-related phenotypes, while *CAG-485* sp002362485 was inversely correlated. Regarding pathway profiles, PWY-7328 (the superpathway of UDP-glucose-derived O-antigen building blocks biosynthesis) was reversely associated with both LOAD and AD proxy, while PWY0-1061 (the superpathway of L-alanine biosynthesis) was positively correlated with CSF Aβ42 levels and reversely associated with CSF p-tau levels, suggesting a potential link to reduced AD risk. Conversely, GLCMANNANAUT-PWY (the superpathway of N-acetylglucosamine, N-acetylmannosamine, and N-acetylneuraminate degradation) was reversely associated with AD proxies and positively correlated with CSF p-tau levels as expected.

Gut microbiota inversely associated with LOAD, AD proxy, and CSF p-tau, or positively associated with CSF Aβ42, were defined as “AD-protective,” whereas those showing the opposite pattern were defined as “AD-risk” (Fig. S2A). “AD-protective” taxa were predominantly mucin degraders and butyrate producers such as *Akkermansia muciniphila B* and *Bifidobacterium species*. In contrast, AD-risk taxa were linked to cardiovascular disease (Fig. S2A).

### Identification of AD-associated circulating biomarkers

A total of 59 circulating biomarkers were significantly linked to AD-related phenotypes. Notably, several BBB-related biomarkers, such as cation-independent mannose-6-phosphate receptor, neutrophil collagenase, and potassium voltage-gated channel subfamily E member 2, along with immune-related biomarkers like FSC-A on plasmacytoid dendritic cell and HLA DR on myeloid dendritic cell, were significantly associated with LOAD and AD proxy (Table S1). Lipid traits, including LDL cholesterol (β = 0.199, *P* < 0.0001) and total cholesterol minus HDL-C (β = 0.17, *P* = 0.0011), were positively correlated with CSF p-tau levels, while remnant cholesterol (non-HDL, non-LDL cholesterol) was reversely correlated with AD proxy (β = −0.26, *P* < 0.001). Additionally, amino acids like glutamine (β = −0.16, *P* = 0.002) and isoleucine (β = −0.16, *P* = 0.002) were significantly associated with LOAD (Fig. 2B).

To assess potential confounding, we compared genome-wide significant SNPs for diet (*n* = 1055) and smoking (*n* = 373) from the GWAS Catalog with the IVs for 69 gut microbial and 64 circulating biomarker traits associated with AD phenotypes. No overlap was detected, thereby supporting the validity of the MR independence assumption (Fig. S3).

We categorized these circulating biomarkers into proteins (including immune and BBB-related biomarkers) and metabolites to assess their functional roles. AD-protective proteins were enriched in PD-1-related signaling pathways, whereas AD-risk proteins were linked to Aβ complexes, such as the APP-Abeta42-VDAC1 complex (Fig. S2B). Enrichment analysis revealed that AD-protective metabolites were involved in pathways like alanine, aspartate, and glutamate metabolism, glyoxylate and dicarboxylate metabolism, and the citrate cycle (adjusted *P* < 0.05, Table S2). In contrast, AD-risk metabolites were linked to the degradation of branched-chain amino acids (BCAA) (adjusted *P* < 0.05, Table S2).

### Prioritizing the role of key features of gut microbiota in AD development

**Fig. 3:**
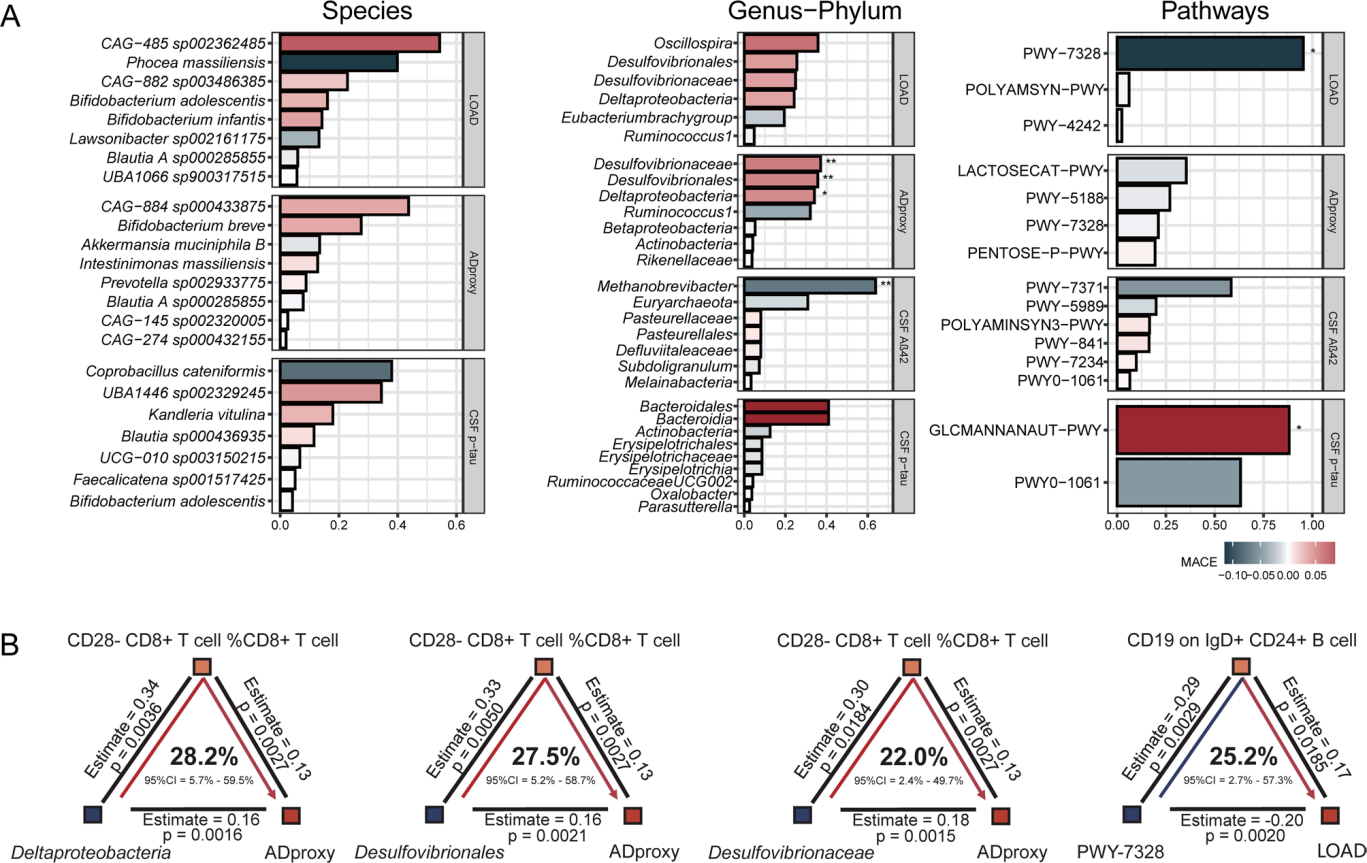
Identification of key gut microbiota and pathways to related AD risk (**A**) and mediation analyses quantifying the effects of key gut microbiota on AD phenotypes via circulating biomarkers (**B**). The bar plot shows key gut microbiota significantly associated with each AD phenotype, as identified through Mendelian randomization Bayesian model averaging (MR-BMA) (no single species was significantly associated with cerebrospinal fluid (CSF)Aβ42 levels). Significance levels are indicated as *P < 0.05; **P < 0.01; ***P < 0.001

Given the strong correlations within gut microbiota at both taxonomic and functional levels, as well as within each category of circulating biomarkers, we conducted MR-BMA analyses using harmonized SNP data to identify causal risk factors for AD outcomes [41]. Six gut microbial features were prioritized in the MR-BMA analysis for their associations with AD-related phenotypes (Table S3-S5). At the species level, no individual species showed a significant association with AD-related outcomes, suggesting that the causal link between gut microbiota and AD likely involves the synergistic effect of multiple species (Fig. 3A). However, at the taxonomic levels, the *Deltaproteobacteria*-*Desulfovibrionales*-*Desulfovibrionaceae* lineage was significantly positively associated with AD proxy (posterior probability (PP) = 0.2, marginal inclusion probability (MIP) = 0.34 ~ 0.37, all adjusted *P* < 0.05), while *Methanobrevibacter* was reversely associated with CSF Aβ42 levels (PP = 0.4, MIP = 0.64, adjusted *P* < 0.01). Regarding pathways, PWY − 7328 (the superpathway of UDP-glucose-derived O-antigen building blocks biosynthesis) was reversely associated with LOAD (PP = 0.2, MIP = 0.95, adjusted *P* = 0.01), and GLCMANNANAUT − PWY (the superpathway of N-acetylglucosamine, N-acetylmannosamine, and N-acetylneuraminate degradation) was positively associated with CSF p-tau levels (PP = 0.4, MIP = 0.88, adjusted *P* < 0.05, Fig. 3A, Table S3, S4). Compared with gut microbial features, the associations of immune, BBB, and metabolic biomarkers with AD risk appeared comparatively weaker (Table S5). Sensitivity analyses showed that varying the prior probability (PP = 0.01–0.3) altered posterior model probabilities but not marginal inclusion probabilities, and changing the prior variance (σ² = 0.01–0.49) did not affect the ranking, ensuring the robustness of the MR-BMA results (Table S6).

We additionally performed UVMR and mediation analyses to evaluate the indirect effects of gut microbiota on AD outcomes via the immune system, BBB-related, and metabolic biomarkers (Table S7, S8). We focused on six features of gut microbiota significantly associated with AD outcomes in MR-BMA analyses. The mediated ratio was estimated at ~ 25%, with circulating biomarkers mediating part of the effect of gut microbes on AD (Table S8). Using the product method, we estimated the indirect effects, calculating standard errors (SE) and confidence intervals (CI) via the delta method. The proportions of the mediation effect of *Deltaproteobacteria*-*Desulfovibrionales*-*Desulfovibrionaceae* lineage on AD proxy via CD28^−^ CD8^+^ T cell %CD8^+^ T cell ranged from 22.0% to 28.2%. Similarly, the mediation ratio of PWY-7328 on LOAD via CD19 on IgD^+^ CD24^+^ B cells was 25.2% (Fig. 3B, Table S8).

### Identification of AD-regulated features of gut microbiota

**Fig. 4:**
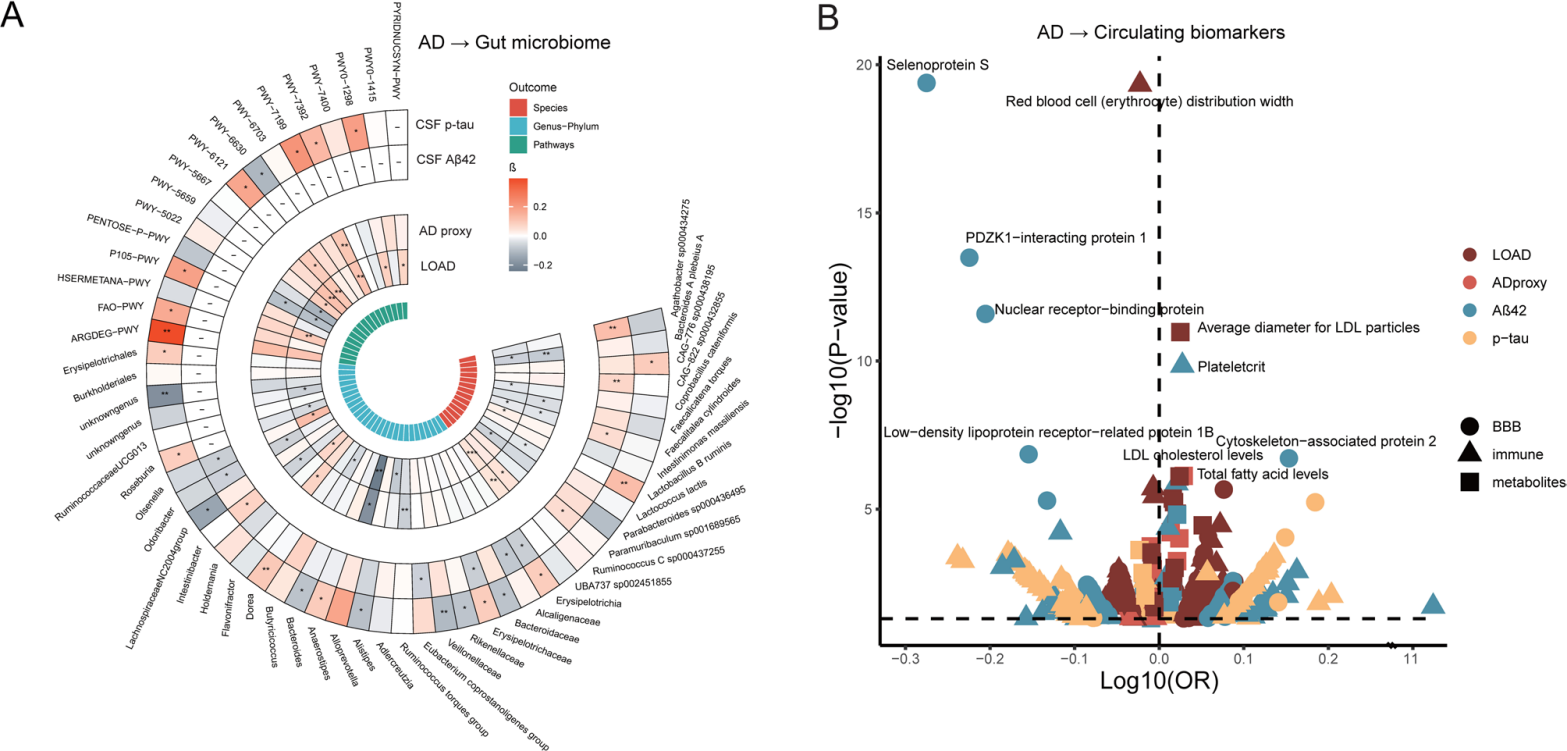
Causal associations between Alzheimer’s disease (AD) phenotypes, circulating biomarkers, and gut microbiota (AD → Gut microbiome/circulating biomarkers). The circular heatmap (**A**) illustrates causal relationships identified in the primary Mendelian randomization analysis using constrained maximum likelihood and model averaging (cML-MA-BIC), showing associations between AD phenotypes and gut microbiota. The inner circle displays associations with late-onset AD (LOAD) and AD proxies, while the outer circle highlights associations with cerebrospinal fluid (CSF) pathological biomarkers. Cells are colored by beta coefficients: red for positive and blue for reverse associations. Gut microbiota features are organized by species, genus-phylum, and metabolic pathways. Significance levels are indicated as *P < 0.05; **P < 0.01; ***P < 0.001. The volcano plot (**B**) displays causal relationships between AD and circulating biomarkers, with dot shapes denoting exposure categories and colors representing different AD phenotypes as outcomes. The top 10 significant associations for circulating biomarkers are labeled. Dashed lines indicate *P*-value (*P* < 0.05) and odds ratio (OR = 1) thresholds

Given the dynamic communication between the gut microbiota and brain via the gut-brain axis, we further performed MR analyses to assess how AD impacts the gut microbiota (Fig. 4A, Table S9). A total of 323 circulating biomarkers and 19 gut microbial features were identified as being associated with AD-related phenotypes (Table S9). At the genus-to-phylum levels, the *Erysipelotrichia*-*Erysipelotrichales*-*Erysipelotrichaceae* lineage was reversely associated with CSF Aβ42 levels and positively associated with CSF p-tau levels, indicating an increase in abundance as AD pathology worsens. At the species level, *Bacteroides A plebeius A* and *UBA737 sp002451855* were reversely associated with LOAD and AD proxy. Similarly, *Faecalitalea cylindroides* was reversely linked to LOAD and AD proxy but positively correlated with CSF Aβ42 levels, suggesting its abundance is inversely related to AD progression. In terms of gut microbiota pathways, PWY-7328 (the superpathway of UDP-glucose-derived O-antigen building blocks biosynthesis) was positively associated with LOAD and CSF p-tau levels. Additionally, PWY-5188 (tetrapyrrole biosynthesis I from glutamate) and POLYAMINSYN3-PWY (superpathway of polyamine biosynthesis II) were positively associated with LOAD, AD proxy, and CSF p-tau levels. Notably, AD-associated microbiota taxa and pathways were linked to short-chain fatty acids (SCFA) production. For example, *Faecalitalea cylindroides*, SCFA producers, and pathways like PWY-5022 (4-aminobutanoate degradation V) were involved. Pathways such as PWY-6703 (preQ0 biosynthesis) and PENTOSE-P-PWY (pentose phosphate pathway) related to preQ0 enzyme and energy metabolism were also identified.

Similarly, from AD to gut microbiota, gut microbiota reversely associated with LOAD, AD proxy, and CSF p-tau levels or positively associated CSF Aβ42 levels were classified as “AD-decreased” taxa, while those with the opposite pattern were regarded as “AD-increased” taxa (Fig. S2A). Enrichment analysis revealed that AD-decreased taxa were associated with healthy lifestyles like Omega-3 supplementation and alkaline drinking water, while AD-increased taxa were linked to diets rich in fructo-oligosaccharides and shiitake mushrooms (Table S10). Both taxa types were related to diseases such as non-alcoholic fatty liver disease and liver cirrhosis (Fig. S2A).

### Identification of AD-regulated circulating biomarkers

We identified 284 significant associations between circulating biomarkers and AD-related phenotypes (Fig. 4B, Table S11). Among the top 10 associations, immune system and BBB-related biomarkers (e.g., selenoprotein S, red blood cell distribution width, PDZK1-interacting protein 1) were inversely associated with AD pathology, while lipid traits like LDL cholesterol and total fatty acid levels were positively linked to AD phenotypes. Interestingly, enrichment analysis showed that the AD-increased and -decreased proteins were enriched in the intestinal immune network for IgA production, except for those general immune-related pathways. AD-increased metabolites were also enriched in pathways like fatty acid biosynthesis, neomycin, kanamycin, and gentamicin biosynthesis (Fig. S2, Table S10).

### Exploring pathways of AD modulation in gut microbiota and shared biological mechanisms

**Fig. 5:**
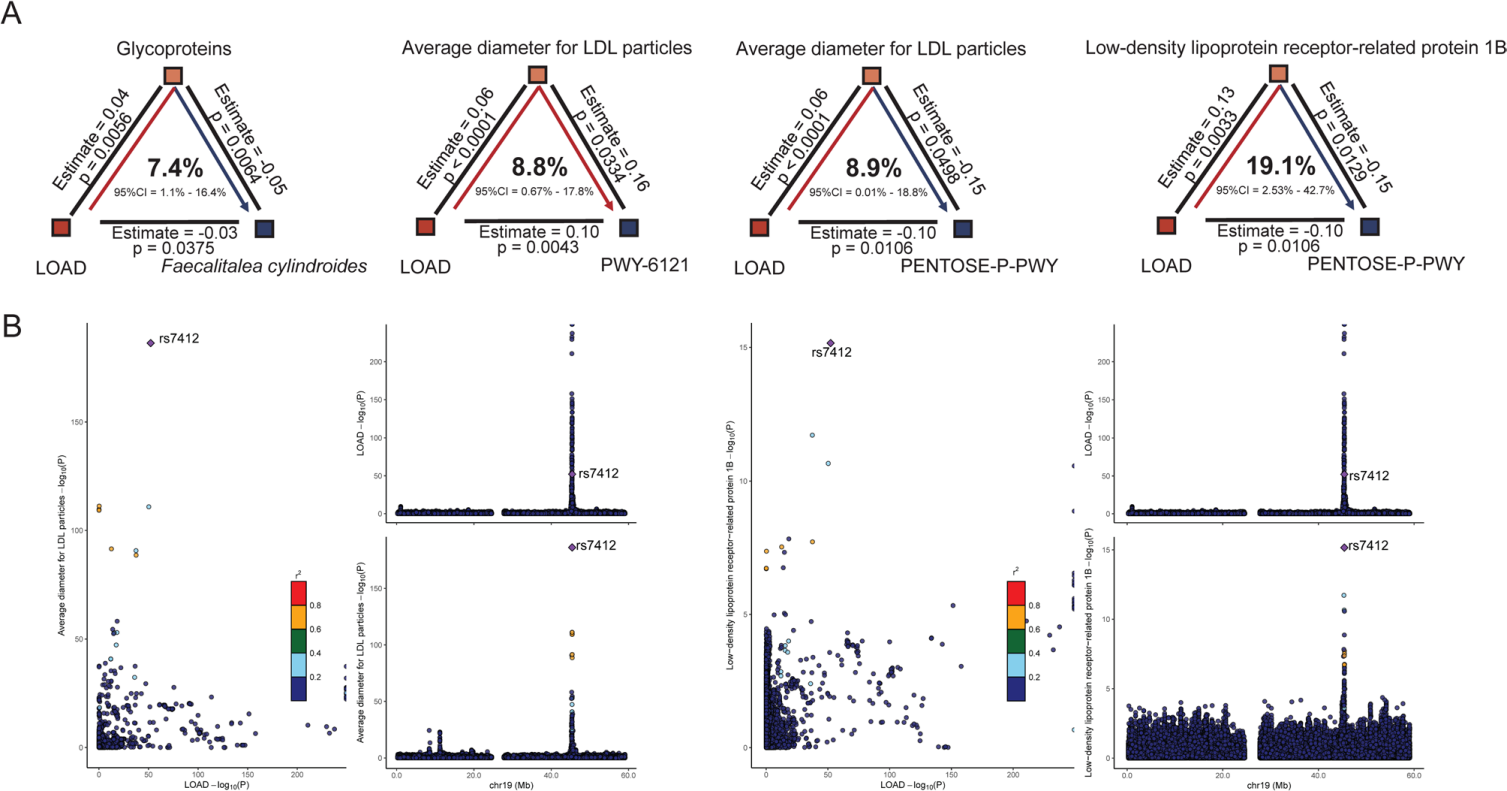
Identification of key mediators between Alzheimer’s disease (AD) phenotypes and gut microbiota, with estimation of mediation effects through mediation analysis (**A**) and colocalization analysis (**B**). Based on univariable Mendelian randomization findings, four features were significantly associated with multiple AD phenotypes, suggesting their potential importance to AD. Mediation analysis identified these mediators in relation to AD phenotypes using a two-step mendelian randomization approach. Colocalization analysis further validated the MR findings, indicating that the average diameter of low-density lipoprotein (LDL) particles and low-density lipoprotein receptor-related protein 1B share causal variants with late-onset AD (LOAD)

To identify robust links between gut microbiota and AD, we focused on five key gut microbiota features (e.g., *Faecalitalea cylindroides*, *UBA737 sp002451855*, *Bacteroides A plebeius A*, PWY-6121, and PENTOSE-P-PWY) that were consistently associated with multiple AD phenotypes (Fig. 4A). Several glycoproteins and LDL-related traits mediated the effects of LOAD on gut microbiota, with mediated ratios ranging from 0.05 to 0.20 (Table S12). For example, glycoproteins mediated 7.4% of the effect on *Faecalitalea cylindroides*, while the mediation effect of LDL particle diameter on PWY-6121 was 8.8%. The mediation ratio of PENTOSE-P-PWY via the low-density lipoprotein receptor-related protein 1B accounted for 19.1% (Fig. 5A, Table S12). Additionally, colocalization analysis indicated that rs7412 is a shared genetic variant between LOAD and the average diameter of LDL particles, as well as in low-density lipoprotein receptor-related protein 1B, with colocalization PP = 1.0 and rs7412 explaining 100% of the posterior contribution (Fig. 5B, Table S13).

### Feedback circuits in the crosstalk between gut microbiota and AD

**Fig. 6:**
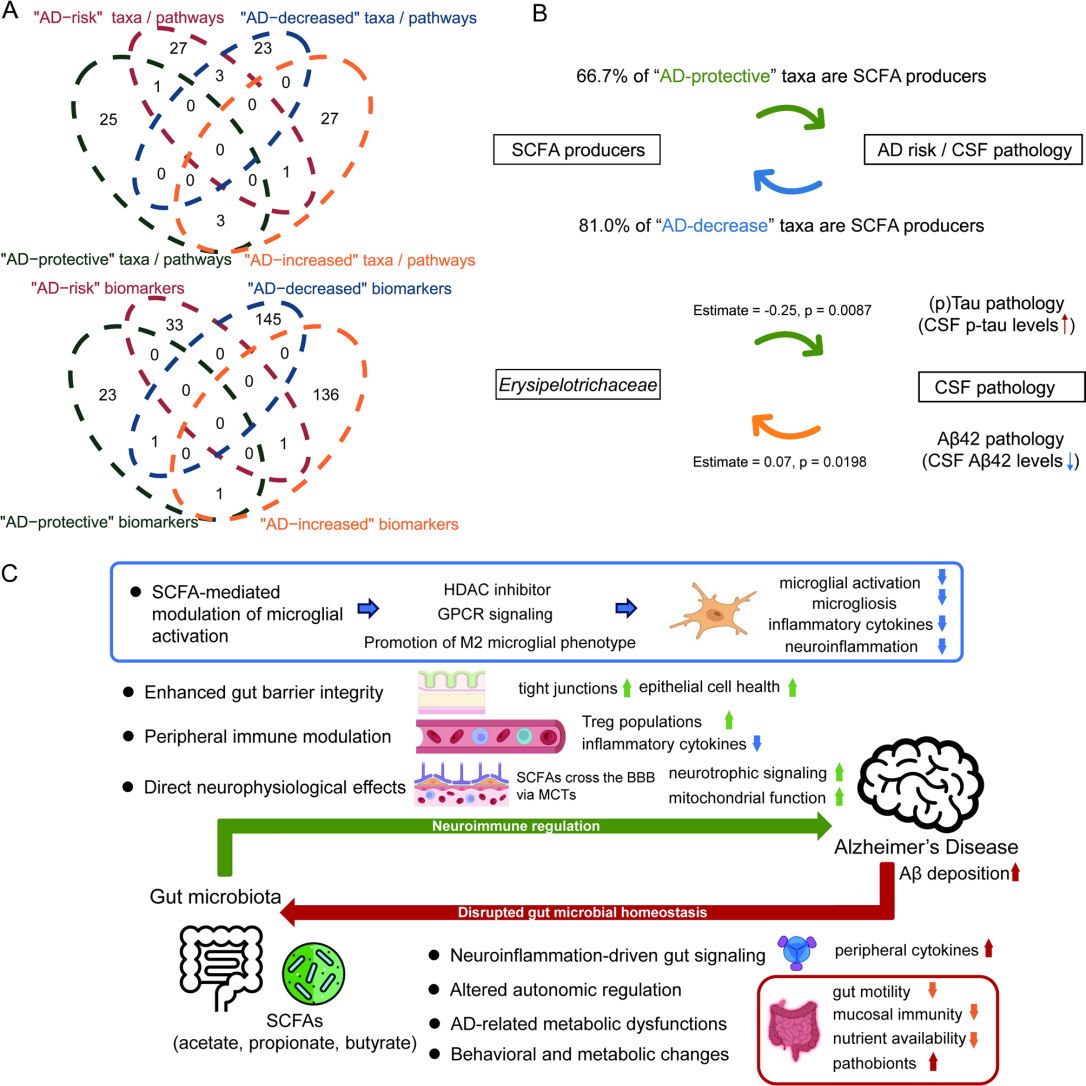
Significant gut microbiota features and immune, BBB-related, metabolic biomarkers identified in the bidirectional Mendelian randomization analyses (**A**); schematic of the potential feedback loop between gut microbiota and Alzheimer’s disease (**B**); and proposed molecular mechanisms within this bidirectional feedback loop (**C**). Effect estimates and P values are derived from the primary Mendelian randomization analysis. AD: Alzheimer’s disease, Aβ: Amyloid-beta, CSF: cerebrospinal fluid, GPCR: G protein–coupled receptor, HDAC: histone deacetylase, SCFA: short-chain fatty acid

We identified the bidirectional feedback loop within the microbiota-gut-brain axis based on previous MR analyses using the Venn plots (Fig. 6A-B). There were 8 microbial taxa and 3 immune biomarkers (HLA DR^+^ CD8^+^ T cell absolute count, CD25 on IgD^+^ CD24^−^ B cell, and CD3 on effector memory CD8^+^ T cell) bidirectionally associated with AD (Fig. 6A). Notably, SCFA-producing taxa exhibited a dual dominance in both “AD-protective” and “AD-decrease” groups (66.7% vs. 81.0%, Table S14, Fig. 6B). In addition, genetically proxied *Erysipelotrichaceae* abundance showed an inverse causal association with CSF p-tau levels (β = −0.25, *P* = 0.0087), while reduced CSF Aβ42 levels exerted a positive causal effect on *Erysipelotrichaceae* abundance (β = 0.07, *P* = 0.0198) (Fig. 6B). In addition, drawing on previous animal experiments and clinical studies, we further illustrated the bidirectional interactions between SCFA-producing gut microbes and AD pathology, encompassing both potential protective mechanisms and AD-induced microbial disruption (Fig. 6C).

While no shared genetic variants were identified linking *Erysipelotrichaceae* abundance to CSF Aβ42 or p-tau levels, colocalization analyses revealed convergent genetic architecture between amyloid and tau pathology at locus rs71352238 (colocalization PP = 1.0, contribution proportion = 100%; Table S15, Fig. S4). Molecular QTL annotation based on the GTEx project showed that rs71352238 is a splicing QTL for *TOMM40* in the cerebellum and an eQTL for *BCAM*, *TOMM40*, and *CTB-171A8.1* across multiple tissues, including brain. The SNP rs7412 was identified as an eQTL for *APOE* in liver, spleen, and cortex (Table S16, Fig. S5). These findings are consistent with the colocalization results.

## Discussion

In this study, we conducted bidirectional two-sample MR and colocalization analyses to investigate the hypothesis that immune signaling, BBB integrity, and systemic metabolism contribute to the bidirectional crosstalk between gut microbiota and AD. To mitigate potential prodromal and selection biases [23], we incorporated comprehensive GWAS summary data covering gut microbiota taxonomy (from phylum to species levels) and pathway profiles, immune system, BBB-related and metabolic circulating biomarkers, as well as LOAD, AD proxy diagnoses, and AD pathological biomarkers. Additionally, we applied multiple MR methods to ensure robust causal relationships. We found that gut microbiota involved in maintaining intestinal barrier function, such as *Akkermansia muciniphila B*, may reduce AD risk, while microbes associated with cardiovascular disease seem to promote AD development. Additionally, circulating biomarkers, including proteins from the PD-1/PD-L1 checkpoint pathway and LDL-related traits, showed genetic associations with AD. As AD progresses, SCFA producers and branched-chain amino acids may be suppressed, while the abundance of liver disease-related microbes, glycoprotein biosynthesis in the gut microbiota, and elevated levels of glycoprotein acetyls, polyunsaturated fatty acids (PUFAs), and other lipids in the blood may increase. Our integrative analyses pinpoint critical microbial mediators and neuroimmune pathways in AD pathogenesis. *Desulfovibrionaceae* and *Methanobrevibacter* emerged as key microbial drivers, whereas *Erysipelotrichaceae* inversely associated with CSF p-tau levels but suppressed by Aβ42, suggesting a pathogenic feedback loop. Mediation analyses implicated CD28^+^CD8^+^ T cells, CD19^+^IgD^+^CD24^+^ B cells, glycoproteins, and LDL in gut-brain crosstalk. Colocalization analyses confirmed shared causality between LOAD and LDL metabolism at rs7412 (PP = 1.0), and revealed rs71352238 (PP = 1.0) as a pleiotropic locus for amyloid-tau copathology.

The progression of AD is a multifaceted, continuous process involving various pathophysiological changes. For individuals at risk of AD (AD proxy) and those with CSF pathologies, we found that gut microbiota associated with improved intestinal barrier function—such as mucin-degrading bacteria and SCFA producers like *Akkermansia muciniphila B* and *Bifidobacterium* spp.—may have protective effects against AD. In contrast, cardiovascular disease-related microbes were associated with an increased risk of AD. Additionally, some gut microbiota showed diverse associations with AD phenotypes indicating that microbes may be differentially distributed across the stages or manifestations of AD. For example, *Bifidobacterium adolescentis* demonstrated a reverse association with CSF p-tau levels but positive associations with LOAD and AD proxy (from gut microbiota to AD), while as AD processing, it showed a reverse association with both LOAD and AD proxy (from AD to gut microbiota). Although taxonomic overlap between gut microbes mediating and responding to AD is limited, functional analysis reveals convergence: 66.7% of “AD-protective” and 81.0% of “AD-decreased” taxa are SCFA producers. SCFAs, including butyrate and propionate, maintain BBB integrity, modulate neuroinflammation, and regulate microglia, exerting neuroprotective effects [42]. Notably, *Lawsonibacter sp002161175* exhibited the strongest effect size among the SCFA-producing species [43], while its relevance to AD pathogenesis need to further investigate. By comparison, *Akkermansia muciniphila* appears more biologically plausible, as it restores gut barrier integrity and modulates immunity in AD models [44, 45], highlighting its potential as a target for microbial-based interventions, including fecal microbiota transplantation (FMT) [46]. These results indicate that functional convergence, rather than taxonomic overlap, underlies gut-brain interactions in AD. Also, while associations did not consistently replicate across all AD phenotypes, such variation may reflect biologically meaningful, stage-specific mechanisms rather than simple inconsistency [47]. These findings provide important insights into the complexity of the gut–brain axis in AD and point to opportunities for tailored interventions across different disease stages, although further research is needed to validate and extend these observations. Previous studies have found its positive association with better cognitive function [48]. Among circulating biomarkers, proteins from the PD-1/PD-L1 checkpoint pathway, as well as glutamine and remnant cholesterol levels, were reversely associated with AD risk. However, Aβ pathology-related proteins and lipoproteins were positively associated with a higher risk of AD. These findings are consistent with previous animal and cellular studies demonstrating the protective effects of both mucin-degrading bacteria [49, 50] and PD-L1/PD-1 checkpoint pathway blockade [51, 52]. Specifically, mucin-degrading bacteria enhance tight junction-related protein expression, promote early bacterial colonization, and attenuate inflammatory responses [49, 50]. Furthermore, PD-L1 blockade in 5XFAD mice ameliorates cognitive deficits and cerebral pathology, while modulation of the PD-L1/PD-1 pathway regulates hippocampal neuronal excitability and modulates learning and memory processes [51, 52].

As the increased risk of AD, there could be a decline in the abundance of SCFA producers (e.g., *Akkermansia muciniphila B*, *Lawsonibacter sp002161175* and *Bifidobacterium* spp.) and pathways involved in butyric acid production (PWY-5022) and preQ0 biosynthesis (PWY-6703). Meanwhile, the increased abundance of microbes associated with liver disease, such as *Erysipelotrichaceae*, may suggest underlying mechanisms contributing to the co-occurrence of diseases. Similarly, circulating levels of BCAA, lactate, docosahexaenoic acid, HDL, and Apolipoprotein A1 (APOA1) decrease, while levels of fatty acid, PUFA, and LDL cholesterol levels rose (Table S5). These findings are consistent with animal studies linking gut microbiota to AD pathologies and cognitive disorders through PUFA-associated neuroinflammation [53]. Additionally, AD-regulated circulating biomarkers were enriched in the intestinal immune network for IgA production, indicating that AD may influence gut microbiota through immune modulation (Fig. S2B).

The enrichment analysis revealed that “AD-protective” and “AD-decreased” microbes were predominantly enriched in individuals engaging in regular exercise and omega-3 supplementation, whereas “AD-risk” and “AD-increased” microbes were enriched in those with a Western-style diet and obesity (Figure S2A). In line with previous research, these associations suggest that lifestyle factors may influence AD risk through their modulatory effects on the gut microbiome, highlighting potential avenues for prevention and intervention [54, 55].

Our MR-BMA analysis suggests that gut microbiota act collectively in AD progression, rather than through individual species. The *Deltaproteobacteria*-*Desulfovibrionales*-*Desulfovibrionaceae* lineage, which plays a role in methylamine cycling and trimethylamine N-oxide production, was elevated in participants with subjective memory complaints and positively associated with AD risk [56–58]. Recent evidence suggests that *Desulfovibrionaceae*, including *Desulfovibrio desulfuricans*, generate trimethylamine (TMA) from dietary methylamines, which is converted to trimethylamine N-oxide (TMAO), a metabolite implicated in AD [59]. Elevated TMAO has been observed in cerebrospinal fluid of individuals with cognitive impairment and is associated with tau pathology and neurodegeneration [60]. Experimental studies show that TMAO promotes neuroinflammation, amyloid accumulation, and cognitive decline [61]. Although direct evidence for *Desulfovibrionaceae*-driven brain TMAO accumulation is limited, their metabolic capacity and the neurotoxic effects of TMAO support a potential role of this family in AD pathogenesis via the gut-liver-brain axis. Conversely, *Methanobrevibacter* was reversely associated with CSF Aβ42 levels, consistent with previous studies linking it to cognitive impairment [62]. Interestingly, similar results were found in reverse MR, which could indicate the bidirectional causal relationship between *Methanobrevibacter* and the AD process and the potential role of protective against the disease (Fig. 4A). Key gut microbiota pathways, such as PWY-7328 (involved in O-antigen biosynthesis) and GLCMANNANAUT-PWY (involved in N-acetylglucosamine degradation), were associated with LOAD and CSF p-tau levels, indicating the role of gut microbiota-driven products in AD pathology. The absence of significantly prioritized causal factors for gut microbiota species and circulating biomarkers in the MR-BMA analysis may reflect several limitations, including insufficient GWAS sample size, dynamic or offsetting effects among correlated exposures, disease stage heterogeneity, and the limited capacity of current SNPs to capture the genetic architecture of microbial taxa. Moreover, the observed negative associations may partly result from limited statistical power due to weak genetic instruments (Table S17). Mediation analysis further highlighted the role of senescent CD28^−^ CD8^+^ T cell in the interaction between the *Deltaproteobacteria*-*Desulfovibrionales*-*Desulfovibrionaceae* lineage and AD proxy. These cell show elevated levels of cytotoxic mediators, like perforin and granzyme B, as well as pro-inflammatory cytokines such as IFN-γ and TNFα, causing substantial damage to surrounding tissues in an antigen-nonspecific manner [63]. Meanwhile, CD19^+^ B cells, involved in the PWY-7328 linked to LOAD, which is related to inflammation [64] and show reduced mtDNA levels in early Alzheimer’s, which associates with increased amyloid deposition in animal models [65]. Those immune-system related biomarkers revealed gut dysbiosis-linked neuroinflammation in AD.

In the gut microbiota-AD communication pathway, glycoproteins and LDL-related phenotypes mediated the regulation of LOAD on gut microbiota, including the butyric and lactic acid producer (e.g., *Faecalitalea cylindroides*) [66], vitamin B1 production related pathway (PWY-6121: 5-aminoimidazole ribonucleotide biosynthesis) [67], and Central energic metabolism (PENTOSE-P-PWY: pentose phosphate pathway). Particularly, the abundance of the vitamin B1 biosynthesis pathway (PWY-6121) was elevated in individuals with higher genetic risk for LOAD, potentially reflecting a compensatory microbial response. Given that impaired cerebral thiamine utilization is a known feature of AD, upregulation of microbial thiamine biosynthesis may help maintain central metabolic homeostasis. This hypothesis is supported by clinical evidence suggesting a similar compensatory adaptation involving thiamine metabolism [68]. These circulating biomarkers might directly and indirectly alter the gut environment, completing the regulatory feedback loop between AD and gut microbiota [69]. While no shared causal variants were identified between gut microbiota, circulating biomarkers, and AD-related phenotypes, the rs7412 variant was shared between LOAD and LDL-related traits (average LDL particle diameter and LDL receptor-related protein 1B). rs7412 is a well-characterized SNP within the *APOE* gene, a major genetic risk locus for AD, with a central role in lipid metabolism [70]. In contrast, rs71352238 is located near *TOMM40*, which encodes a core component of the mitochondrial protein import channel. Emerging evidence suggests that the CC genotype of rs71352238 increases AD risk independently of *APOE* and may exert a synergistic effect when co-occurring with the *APOE* ε4 allele [71, 72]. Also, rs7412 was excluded in the UVMR analysis by MR-PRESSO, ensuring the validity of MR assumptions.

The bidirectional MR analysis indicated the potential feedback circuits within the microbiota-gut-AD crosstalk. SCFA-producing bacteria exhibited reduced abundance with AD risk progression via compromised BBB integrity (e.g., glycoprotein-mediated suppression of *Faecalitalea cylindroides*), concomitant with neuroinflammatory cascades, suggesting a self-perpetuating cycle wherein AD pathogenesis amplifies gut dysbiosis [12, 73]. In addition, as a member of SCFA producers (Table S11), *Erysipelotrichaceae* depletion mediated Aβ42-associated p-tau mitigation via gut-brain crosstalk, establishing feedforward amyloidosis-dysbiosis coupling. Previous studies have reported its associations with lower CSF p-tau [74] and an increase in AD patients [75], which is consistent with our finding. Although the colocation analysis only identified the shared variant (rs71352238, *APOE* locus) between CSF Aβ42 levels and p-tau levels, a study also showed the association between another *APOE* locus, rs429358 [76].

Recent evidence from both clinical and animal studies suggests that SCFAs, particularly butyrate, can cross the BBB and may influence AD-related processes. In humans, butyrate has been detected in cerebrospinal fluid [77–79] and has been linked to enhanced BBB integrity and modulation of inflammatory signaling. Consistent with these observations, animal studies further show that SCFA administration can improve cognition, reduce amyloid burden, and promote microglial Aβ clearance [80, 81]. Together, findings from clinical and animal research point to a potential neuroprotective role of SCFAs through BBB penetration and regulation of neuroinflammation. Based on previous animal and clinical studies, we summarized the potential mechanisms underlying the feedback loop between SCFA-producing gut microbes and AD pathology (Fig. 6C). SCFAs exert neuroprotective effects through histone deacetylase (HDAC) inhibition, G protein–coupled receptor (GPCR) signaling, and M2 microglial polarization [82–86], while also enhancing gut barrier integrity, modulating peripheral immunity, and supporting neurophysiological functions [87–89]. Conversely, AD pathology may disturb microbial homeostasis via neuroinflammation-driven cytokine signaling, autonomic dysregulation, metabolic impairments, and behavioral changes [90–94]. In addition, Aβ deposition has been implicated in enteric inflammation, which promotes microbial remodeling and dysbiosis [95, 96]. This bidirectional framework suggests a vicious cycle in which AD pathology exacerbates gut dysbiosis, while impaired SCFA signaling accelerates neurodegeneration, underscoring SCFA-related pathways as potential therapeutic targets.

Although AD pathology aggravates gut dysbiosis, microbiota modulation through probiotics or FMT may help disrupt this cycle and slow progression. In animal models, FMT restored microbial balance, reduced amyloid pathology and neuroinflammation, and improved cognition, partly by enriching SCFA-producing taxa and suppressing pro-inflammatory signaling [97, 98]. Mechanistically, FMT enriches SCFA-producing taxa and downregulates pro-inflammatory pathways such as TLR4/NF-κB [99]. Case reports in AD patients have also noted cognitive benefits [100]. While probiotic interventions generally yield more modest effects, they remain a feasible adjunct strategy [101]. Collectively, these findings highlight the translational potential of targeting the gut–brain axis to protect against neurodegeneration, supported by evidence from preclinical interventions including SCFA supplementation, probiotic administration, FMT, and even LDL-lowering therapies [46, 102–105].

Overall, our study verified the hypothesis that immune signaling, BBB integrity, and systemic metabolism contribute to the bidirectional crosstalk between gut microbiota and AD. Using Mendelian randomization, we leveraged extensive GWAS data to explore these causal links. The strengths of our study include the integration of diverse large-scale GWAS datasets to capture complex phenotypes, particularly for AD CSF biomarkers, and the application of multiple MR methods along with colocalization analysis to yield more robust results. Additionally, enrichment analyses were conducted to elucidate underlying biological mechanisms, providing causal evidence for the relationship between gut microbiota and AD at the population level. However, there are some limitations that should be acknowledged. First, the GWAS summary data on gut microbiota and circulating biomarkers may be limited by rapidly evolving measurement technologies, suggesting that more bacteria or biomarkers could be discovered in the future. Second, since the summary data we used were sourced from a publicly available GWAS database, limiting our ability to assess population stratification, the generalization of the estimation of MR [106], and the effects of potential confounders. Third, heterogeneity across GWAS datasets, including ancestry, age, and profiling methods, may introduce bias; for example, although ~ 78% of MiBioGen participants are of European ancestry [25], the inclusion of non-European samples and the lack of publicly available ancestry-specific data precluded European-only sensitivity analyses, warranting validation in more homogeneous cohorts. Finally, while MR offers a partial causal explanation at the population level, it estimates lifetime exposure rather than modeling specific stages of AD. Although we included diverse AD phenotypes—clinical diagnosis, AD proxy status, and CSF biomarkers reflecting disease stages—a key limitation is that MR provides lifetime average estimates and cannot resolve the temporal sequence of microbiome-AD interactions, which warrants longitudinal and mechanistic studies. Future studies should integrate microbial networks with functional data to better capture community-level interactions by which the gut microbiota may contribute to AD development. Further research involving diverse populations and study designs, such as animal studies and clinical intervention research, is warranted to validate and explore these underlying mechanisms.

## Conclusion

In conclusion, our study represents the first attempt to investigate the hypothesis that immune signaling, BBB integrity, and systemic metabolism contribute to the bidirectional crosstalk between gut microbiota and AD. Our findings highlight gut-brain crosstalk mechanisms involving immune signaling, BBB integrity, and systemic metabolism, which are exacerbated by AD-linked gut dysbiosis characterized by depletion of SCFA-producing taxa, ultimately driving neuropathogenesis through a self-reinforcing inflammatory cycle. These findings illuminate new opportunities for microbiome-targeted interventions (e.g., FMT), precision biomarker development (e.g., CSF p-tau/SCFA ratios), and therapies for AD prevention and treatment.

## Supplementary Information

Supplementary Material 1.

Supplementary Material 2.

Supplementary Material 3.

## Data Availability

All data needed to evaluate the conclusions in the paper are present in the paper and/or the Supplementary Materials. This study used publicly available GWAS summary data at MRbase (https://www.mrbase.org/) and GWAS Catalog ([https://www.ebi.ac.uk/gwas/](https://www.ebi.ac.uk/gwas)). The scripts used in this manuscript can be found at the GitHub repository (https://github.com/JINCHENG-bio/GM-CB-AD).

## Abbreviations

AD: Alzheimer’s disease
Aβ: Amyloid-beta
BBB: Blood-brain barrier
BCAAs: Branched-chain amino acids
CSF: Cerebrospinal fluid
GWAS: Genome-wide association study
GPCR: G protein–coupled receptor
HDAC: Histone deacetylase
IV: Instrumental variables
LDL: Low-density lipoprotein
LOAD: Late-onset AD
MIP: Marginal inclusion probability
MR: Mendelian randomization
MR-BMA: MR-Bayesian model averaging
NFTs: Neurofibrillary tangles
p-tau: Phosphorylated tau
PD-1: Programmed Cell Death Protein 1
PD-L1: Programmed Death-Ligand 1
PP: Posterior probability
QTL: Quantitative trait loci
SCFA: Short-chain fatty acid
UVMR: Univariable MR
