# Supplementary material for: Immune, blood-brain barrier, and metabolic biomarkers mediate gut-brain axis crosstalk in alzheimer’s disease
Source: Biomark Res. 2025 Oct 29;13:137. doi: 10.1186/s40364-025-00851-6 (PMC12573957; doi:10.1186/s40364-025-00851-6)

Supplementary figures

Fig. S1.

The flowchart of the two-sample mendelian randomization process.


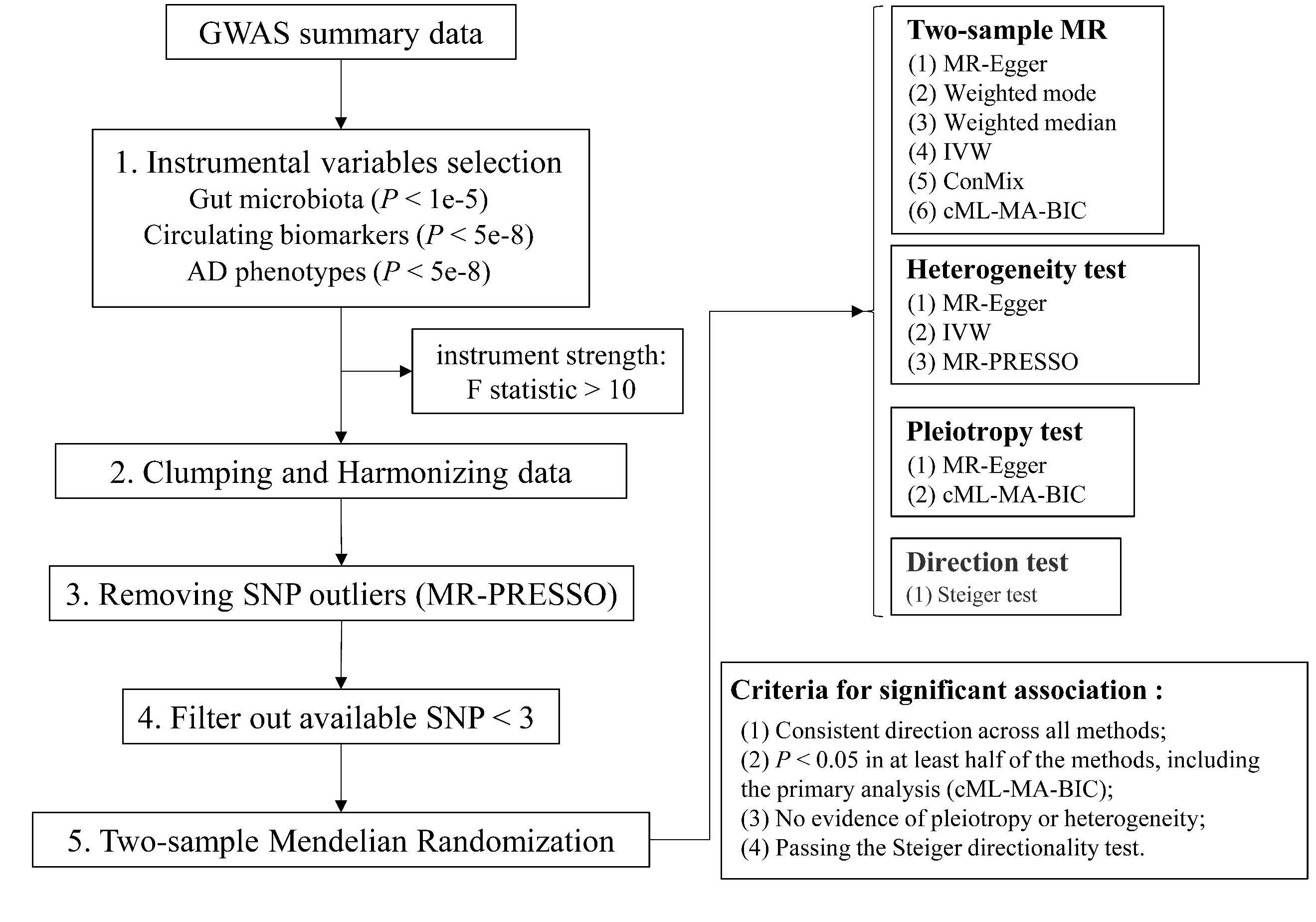


Fig. S2.

The enrichment analysis of AD-related gut microbiota and circulating biomarkers.


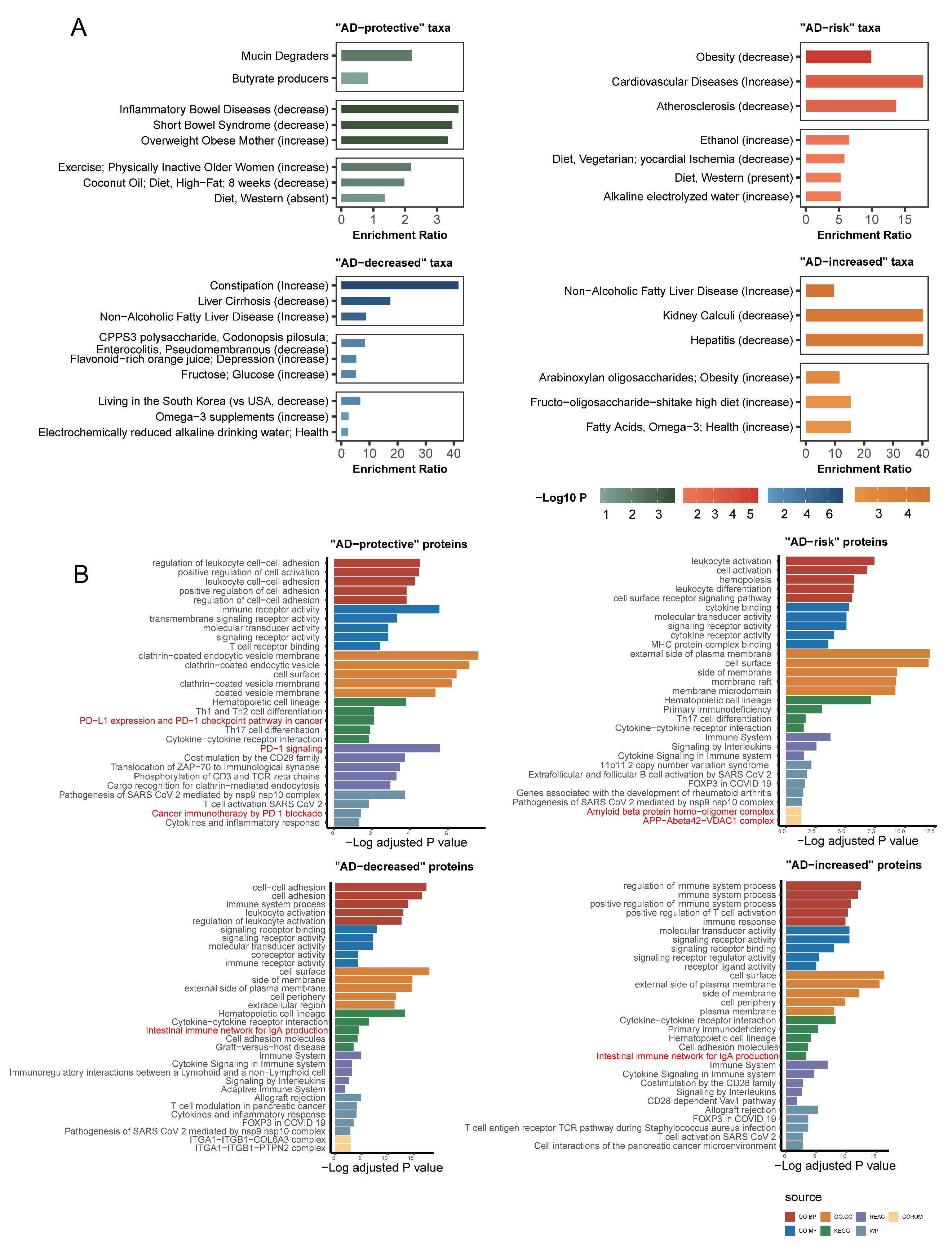


Fig. S3.

Venn plot of instrumental variables for AD-associated gut microbiota features and circulating biomarkers, and instrumental variables for smoking behavior and diet measures from the GWAS Catalog. GM: gut microbiota; CB: circulating biomarker.


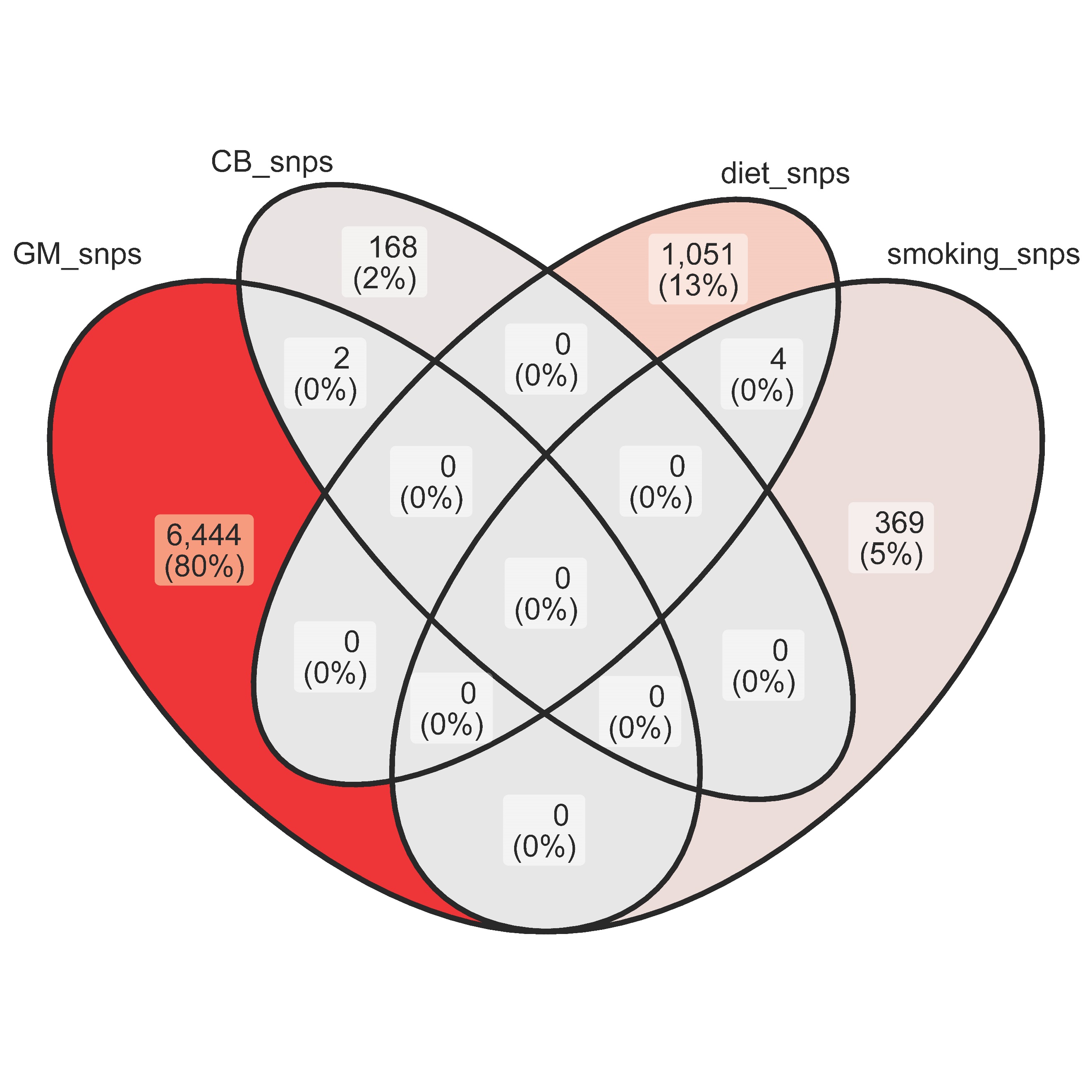


Fig. S4.

Colocalization analysis indicated that the CSF p-tau levels and CSF Aβ42 levels share causal variant rs71352238.
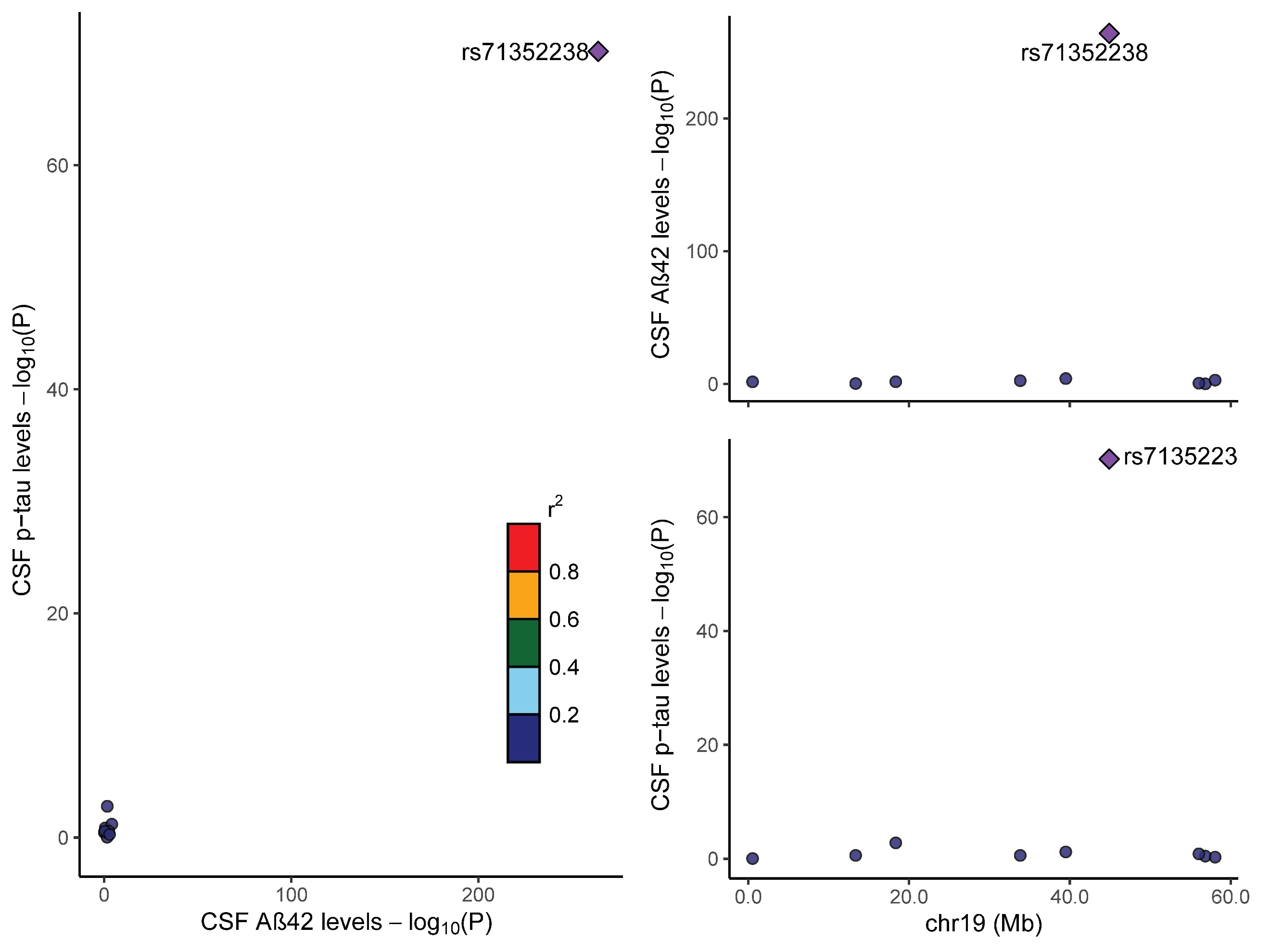


Fig. S5.

Expression of genes associated with rs7412 and rs71352238 in different tissues from GTEx. (A) rs7412-associated gene *APOE*; (B-D) rs71352238-associated genes *BCAM*, *TOMM40*, and *CTB-171A8.1*.


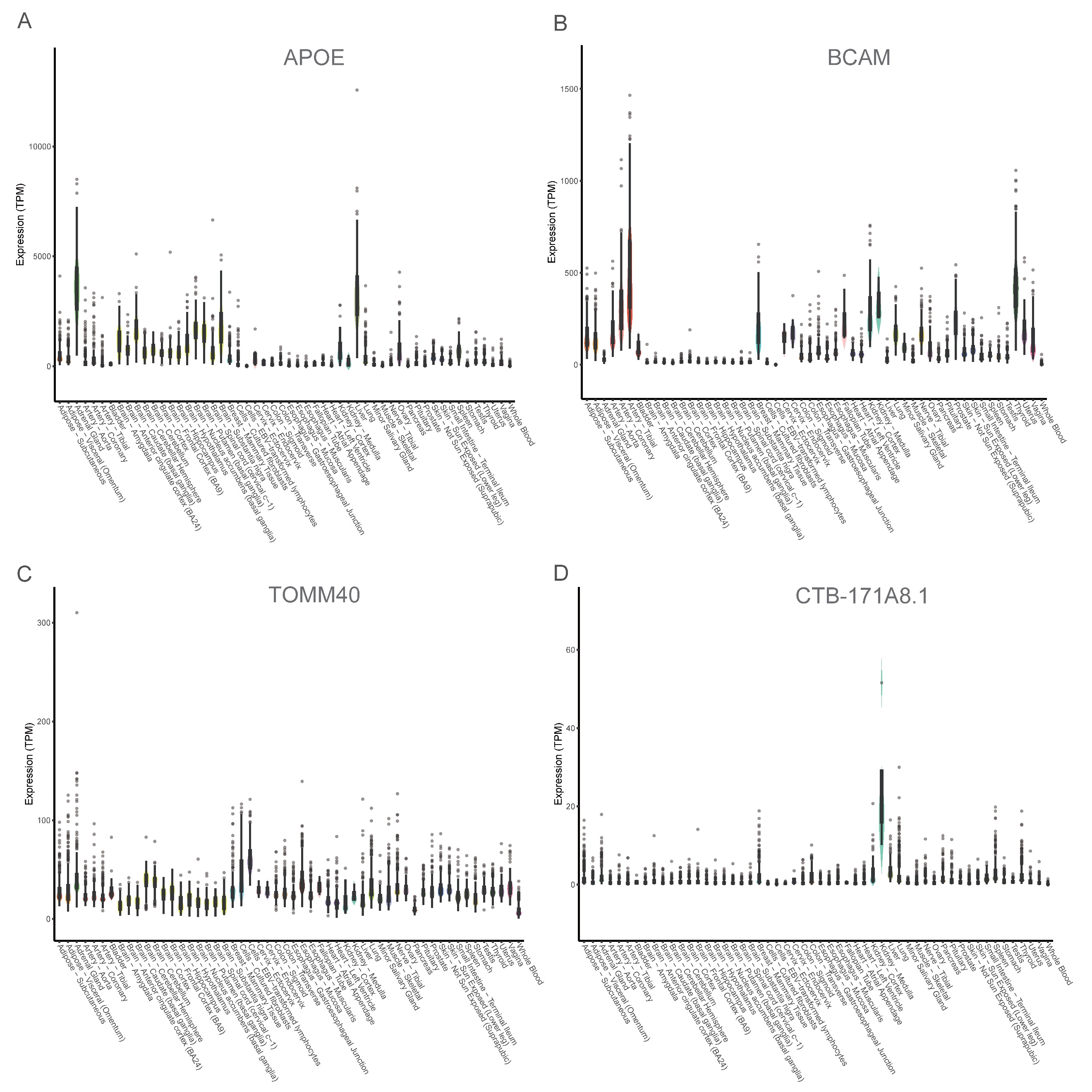

Supplement: Supplementary file 1 — Supplementary Material 1. [file 40364_2025_851_MOESM1_ESM.docx]
