# Supplementary material for: Immune, blood-brain barrier, and metabolic biomarkers mediate gut-brain axis crosstalk in alzheimer’s disease
Source: Biomark Res. 2025 Oct 29;13:137. doi: 10.1186/s40364-025-00851-6 (PMC12573957; doi:10.1186/s40364-025-00851-6)
Supplement: Supplementary file 3 — Supplementary Material 3. [file 40364_2025_851_MOESM3_ESM.docx]

Supplementary method

**GWAS summary data**

**Gut microbiota.** Genetic variants for gut microbiota included both taxonomic and pathway profiles. The taxonomic profiles were derived from two large gut microbiota GWAS published to date: the first was performed by the FINRISK 2002 (FR02) cohort, comprising 5,959 participants and 473 taxa[1]; the other was conducted by the MiBioGen consortium, which included 18,340 participants and 211 taxa[2]. Additionally, pathway profiles were accessed from the Dutch Microbiome project, which involved 7,738 participants and covered 205 pathway features[3].

**Circulating biomarkers.** The GWAS data of immune system and BBB-related traits were sourced from Lindbohm et al. study [4], including 1155 immune-related traits and 699 BBB-related traits, with participants numbers ranging from 3,301 to 563,964. Metabolic data were obtained from a large sample size of 115,082 UK Biobank participants, covering 249 metabolic traits[5].

**AD phenotype.** The GWAS data for AD included both AD diagnosis traits and AD pathological biomarkers. Genetic instruments for LOAD were acquired from the largest meta-analysis for LOAD to date (21,982 cases and 41,944 controls)[6], and that for AD proxy was sourced from a meta-analysis containing the aforementioned LOAD study and GWAS by proxy for a family history of AD in the UK Biobank (53,042 proxy cases—either diagnosed with AD or reporting a parent or sibling with dementia—and 355,900 controls). AD proxy was included to address the limited sample size of LOAD and potential bias arising from the disease stage in case samples[7]. We further included CSF levels of Aβ42 and p-tau as the pathological biomarkers of AD from a meta-analysis with the largest sample size (n = 13,116) so far[8] (supplementary method). All GWAS summary data were sourced from European population.

Since the GWAS summary data of CSF Aβ42 levels and CSF p-tau levels only contain Z score, we derived the estimate of the SNP ($\beta$) and the standard error (*SE*) from Z score and the MAF of significant QTL, using the following formulae[9,10]:

$$\beta=z{(\surd((2p(1-p)(n+z^2))))}^{-1}$$

$$SE={(\sqrt{(2p(1-p)(n+z^{2}))})}^{-1}$$

where p is the minor allele frequency (MAF), n is the sample size, and z is the meta-analysis Z score.

**Instrumental variable selection**

The selection of IV was based on statistical associations (Fig. S1). First, single nucleotide polymorphisms (SNPs) significantly associated with gut microbiota taxa ($P < 1\times{10}^{-5}$)[11] or Immune system, BBB-related, and metabolic biomarkers ($P < 5\times{10}^{-8}$) were included. SNPs with a minor allele frequency $\leq0.01$ were removed. Linkage disequilibrium clumping was then applied with a stringent threshold ($R^{2} <0.001$ in the 1000 Genomes European reference panel within 10 Mb windows) to retain independent IVs with the lowest P values for taxa and biomarkers. When no shared SNPs existed between exposure and outcome, proxy SNPs ($R^{2} >0.8$) from the 1000 Genomes European reference panel were used.

F-statistics were calculated for each exposure, with values greater than 10 indicating sufficient instrument strength[12]. The F-statistic was derived from the variance explained (R^2^) by SNPs for each exposure by $\frac{R^{2}\times(N-1-K)}{(1-R^{2})\times K}$, where K is the number of SNPs and N the sample size. Additionally, $I_{GX}^{2}$ was calculated to assess the expected relative bias (or dilution) due to measurement error.

We compared genome-wide significant SNPs for diet and smoking from the GWAS Catalog with the IVs for gut microbial and circulating biomarker traits that showed significant associations with AD phenotypes, and did not identify any overlap (Fig. S3).

**Univariable Mendelian randomization and sensitivity analyses**

As different Mendelian randomization methods are based on different assumptions, the adoption of multiple Mendelian methods helps minimize false positives. Multiple UVMR methods were performed in the present study including inverse variance weighted (IVW), MR-Egger regression, weighted median, weighted model, Mendelian randomization pleiotropy residual sum and outlier (MR-PRESSO), Contamination mixture method (ConMix) and constrained maximum likelihood and model averaging (cML-MA). IVW offers unbiased estimates when no invalid IVs are present[13], while MR-Egger assumes instrument strength independent of direct effects (InSIDE), providing reliable estimates if most IVs are valid[14]. The weighted median remains consistent even with up to 50% invalid IVs, and the weighted mode has more power and less bias than MR-Egger under InSIDE violations[15]. MR-PRESSO addresses horizontal pleiotropy by removing outliers, though it requires around 50% valid IVs and the InSIDE assumption[16]. ConMix[17] and cML-MA[18] are robust across varied scenarios, with ConMix identifying different causal pathways for the same exposure, and cML-MA accounting for pleiotropic IVs, population stratification, and subject overlap. cML-MA-BIC, with its superior power and error control, served as the primary analysis[18].

To explore pleiotropy and heterogeneity, we performed sensitivity analyses, including the MR-Egger intercept test, Cochran’s Q (for IVW and MR-Egger), and “leave-one-out” analysis to detect SNP heterogeneity. The Steiger directionality test was used to confirm the causal direction between exposures and outcomes. For the robustness of our findings, we restricted our analysis to harmonized data with at least three IVs. MR-PRESSO was iteratively used to remove pleiotropic outliers ($NbDistribution = 5000, P < 0.05$), and only after outliers were removed were other MR methods applied. Significant associations were identified based on: (1) consistent direction across all methods, (2) *P* < 0.05 in at least half of the methods, including the primary analysis, (3) no evidence of pleiotropy or heterogeneity, and (4) passing the Steiger directionality test (Fig. S1). Duplicate GWAS traits were excluded by retaining only the study with the largest sample size for each trait. For metabolic traits, we further filtered out lipoprotein subclass compositions with uncertain biological relevance to ensure a clear interpretation of metabolic traits[19].

**Enrichment analysis**

To understand the biological processes regulated by the AD-associated exposures, we performed enrichment analyses with MicrobiomeAnalyst[20], MetaboAnalyst[21], and g:Profiler[22] for the gut microbiota taxa, blood metabolites, and proteins (including those bound to blood cells) separately. The *P* value was adjusted with the false discovery rate (FDR), 0.05 was set as significant threshold.

**Mendelian randomization Bayesian model averaging**

MR Bayesian Model Averaging (MR-BMA) extends multivariable Mendelian randomization by incorporating Bayesian variable selection, enabling the identification of true causal risk factors while accounting for correlated exposures[23]. Given the high correlation among features of high-throughput experiments, MR-BMA was used to identify key traits causally linked to Alzheimer’s disease (AD). The Nyholt procedure for effective tests was applied to control the false discovery rate, with a significance threshold set at an adjusted *P* value < 0.05[24]. To assess the robustness of model averaging, we assessed prior sensitivity by varying the prior probability of selecting a risk factor (*P* = 0.01 - 0.3, corresponding to 0.3 - 9.0 expected causal factors) and prior variance (σ² = 0.01 - 0.49)[23].

**Mediation analysis**

After identifying AD-associated features of gut microbiota and circulating biomarkers, UVMR was performed to explore their causal relationships. For features of gut microbiota that linked to both AD and associated biomarkers (i.e., gut microbiota → circulating biomarkers → AD), we performed a mediation analysis using the product method to estimate the total indirect effect as well as the individual indirect effects mediated by each biomarker.

This quantified the direct and indirect effects of exposures on AD, clarifying the mediating role of specific biomarkers[25]. A reverse analysis was also performed to assess the pathway from AD to circulating biomarkers and then to gut microbiota (AD → circulating biomarkers → gut microbiota). The standard error (SE) and confidence interval (CI) of the indirect effects were estimated using the Delta method[25].

**Multi-trait colocalization analyses**

To validate the MR findings, multi-trait colocalization analysis using HyPrColoc (hypothesis prioritization for multi-trait colocalization) was conducted following Mendelian randomization mediation analysis[26]. Variant selection followed the methods described earlier (see Instrumental variable selection of Method section), and all variants across datasets were harmonized to the same effect alleles before conducting the colocalization analysis.

Reference

1. Qin Y, Havulinna AS, Liu Y, Jousilahti P, Ritchie SC, Tokolyi A, et al. Combined effects of host genetics and diet on human gut microbiota and incident disease in a single population cohort. Nat Genet. 2022;54:134–42.

2. Kurilshikov A, Medina-Gomez C, Bacigalupe R, Radjabzadeh D, Wang J, Demirkan A, et al. Large-scale association analyses identify host factors influencing human gut microbiome composition. Nat Genet. 2021;53:156–65.

3. Lopera-Maya EA, Kurilshikov A, van der Graaf A, Hu S, Andreu-Sánchez S, Chen L, et al. Effect of host genetics on the gut microbiome in 7,738 participants of the Dutch Microbiome Project. Nat Genet. 2022;54:143–51.

4. Lindbohm JV, Mars N, Sipilä PN, Singh-Manoux A, Runz H, Livingston G, et al. Immune system-wide Mendelian randomization and triangulation analyses support autoimmunity as a modifiable component in dementia-causing diseases. Nat Aging. 2022;2:956–72.

5. Richardson TG, Leyden GM, Wang Q, Bell JA, Elsworth B, Davey Smith G, et al. Characterising metabolomic signatures of lipid-modifying therapies through drug target mendelian randomisation. PLoS Biol. 2022;20:e3001547.

6. Kunkle BW, Grenier-Boley B, Sims R, Bis JC, Damotte V, Naj AC, et al. Genetic meta-analysis of diagnosed Alzheimer’s disease identifies new risk loci and implicates Aβ, tau, immunity and lipid processing. Nat Genet. 2019;51:414–30.

7. Schwartzentruber J, Cooper S, Liu JZ, Barrio-Hernandez I, Bello E, Kumasaka N, et al. Genome-wide meta-analysis, fine-mapping and integrative prioritization implicate new Alzheimer’s disease risk genes. Nat Genet. 2021;53:392–402.

8. Jansen IE, van der Lee SJ, Gomez-Fonseca D, de Rojas I, Dalmasso MC, Grenier-Boley B, et al. Genome-wide meta-analysis for Alzheimer’s disease cerebrospinal fluid biomarkers. Acta Neuropathol. 2022;144:821–42.

9. Zhu Z, Zhang F, Hu H, Bakshi A, Robinson MR, Powell JE, et al. Integration of summary data from GWAS and eQTL studies predicts complex trait gene targets. Nat Genet. 2016;48:481–7.

10. Võsa U, Claringbould A, Westra H-J, Bonder MJ, Deelen P, Zeng B, et al. Large-scale cis- and trans-eQTL analyses identify thousands of genetic loci and polygenic scores that regulate blood gene expression. Nat Genet. 2021;53:1300–10.

11. Sanna S, van Zuydam NR, Mahajan A, Kurilshikov A, Vich Vila A, Võsa U, et al. Causal relationships among the gut microbiome, short-chain fatty acids and metabolic diseases. Nat Genet. 2019;51:600–5.

12. Burgess S, Thompson SG, CRP CHD Genetics Collaboration. Avoiding bias from weak instruments in Mendelian randomization studies. International Journal of Epidemiology. 2011;40:755–64.

13. Burgess S, Dudbridge F, Thompson SG. Combining information on multiple instrumental variables in Mendelian randomization: comparison of allele score and summarized data methods. Statistics in Medicine. 2016;35:1880–906.

14. Bowden J, Davey Smith G, Burgess S. Mendelian randomization with invalid instruments: effect estimation and bias detection through Egger regression. International Journal of Epidemiology. 2015;44:512–25.

15. Bowden J, Davey Smith G, Haycock PC, Burgess S. Consistent Estimation in Mendelian Randomization with Some Invalid Instruments Using a Weighted Median Estimator. Genetic Epidemiology. 2016;40:304–14.

16. Verbanck M, Chen C-Y, Neale B, Do R. Detection of widespread horizontal pleiotropy in causal relationships inferred from Mendelian randomization between complex traits and diseases. Nat Genet. 2018;50:693–8.

17. Burgess S, Foley CN, Allara E, Staley JR, Howson JMM. A robust and efficient method for Mendelian randomization with hundreds of genetic variants. Nat Commun. 2020;11:376.

18. Xue H, Shen X, Pan W. Constrained maximum likelihood-based Mendelian randomization robust to both correlated and uncorrelated pleiotropic effects. The American Journal of Human Genetics. 2021;108:1251–69.

19. Mora S. Advanced Lipoprotein Testing and Subfractionation Are Not (Yet) Ready for Routine Clinical Use. Circulation. 2009;119:2396–404.

20. Lu Y, Zhou G, Ewald J, Pang Z, Shiri T, Xia J. MicrobiomeAnalyst 2.0: comprehensive statistical, functional and integrative analysis of microbiome data. Nucleic Acids Research. 2023;51:W310–8.

21. Pang Z, Lu Y, Zhou G, Hui F, Xu L, Viau C, et al. MetaboAnalyst 6.0: towards a unified platform for metabolomics data processing, analysis and interpretation. Nucleic Acids Research. 2024;gkae253.

22. Kolberg L, Raudvere U, Kuzmin I, Adler P, Vilo J, Peterson H. g:Profiler—interoperable web service for functional enrichment analysis and gene identifier mapping (2023 update). Nucleic Acids Research. 2023;gkad347.

23. Zuber V, Colijn JM, Klaver C, Burgess S. Selecting likely causal risk factors from high-throughput experiments using multivariable Mendelian randomization. Nat Commun. 2020;11:29.

24. Levin MG, Zuber V, Walker VM, Klarin D, Lynch J, Malik R, et al. Prioritizing the Role of Major Lipoproteins and Subfractions as Risk Factors for Peripheral Artery Disease. Circulation. 2021;144:353–64.

25. Carter AR, Sanderson E, Hammerton G, Richmond RC, Davey Smith G, Heron J, et al. Mendelian randomisation for mediation analysis: current methods and challenges for implementation. Eur J Epidemiol. 2021;36:465–78.

26. Foley CN, Staley JR, Breen PG, Sun BB, Kirk PDW, Burgess S, et al. A fast and efficient colocalization algorithm for identifying shared genetic risk factors across multiple traits. Nat Commun. 2021;12:764.
